# Y-chromosomal analysis of clan structure of Kalmyks, the only European Mongol people, and their relationship to Oirat-Mongols of Inner Asia

**DOI:** 10.1038/s41431-019-0399-0

**Published:** 2019-04-11

**Authors:** Natalia Balinova, Helen Post, Alena Kushniarevich, Rodrigo Flores, Monika Karmin, Hovhannes Sahakyan, Maere Reidla, Ene Metspalu, Sergey Litvinov, Murat Dzhaubermezov, Vita Akhmetova, Rita Khusainova, Phillip Endicott, Elza Khusnutdinova, Keemya Orlova, Elza Bakaeva, Irina Khomyakova, Nailya Spitsina, Rena Zinchenko, Richard Villems, Siiri Rootsi

**Affiliations:** 1 Federal State Budgetary Institution Research Centre for Medical Genetics, Moscow, 115522 Russia; 2 Estonian Biocentre, Institute of Genomics, University of Tartu, Tartu, 51010 Estonia; 3 Department of Evolutionary Biology, Institute of Molecular and Cell Biology, University of Tartu, Tartu, 51010 Estonia; 4 Laboratory of Ethnogenomics, Institute of Molecular Biology of National Academy of Sciences, Yerevan, 0014 Armenia; 5 Institute of Biochemistry and Genetics - Subdivision of the Ufa Federal Research Centre of the Russian Academy of Sciences, Ufa, 450054 Russia; 6 Department of Genetics and Fundamental Medicine, Bashkir State University, Ufa, 450076 Russia; 7 The UMR 7206, Muséum National d’Histoire Naturelle, Site du Musée de l’Homme, Paris, 75116 France; 8 Institute of Oriental Studies, Russian Academy of Sciences, Moscow, 107031 Russia; 9 Kalmyk Scientific Center, Russian Academy of Sciences, 358000 Elista, Russia; 10 Anuchin Institute and Museum of Anthropology, Lomonosov Moscow State University, Moscow, 125009 Russia; 11 Institute of Ethnology and Anthropology, Russian Academy of Sciences, Moscow, 119991 Russia

**Keywords:** Genetics, Population genetics

## Abstract

Kalmyks, the only Mongolic-speaking population in Europe, live in the southeast of the European Plain, in Russia. They adhere to Buddhism and speak a dialect of the Mongolian language. Historical and linguistic evidence, as well a shared clan names, suggests a common origin with Oirats of western Mongolia; yet, only a limited number of genetic studies have focused on this topic. Here we compare the paternal genetic relationship of Kalmyk clans with ethnographically related groups from Mongolia, Kyrgyzstan and China, within the context of their neighbouring populations. A phylogeny of 37 high-coverage Y-chromosome sequences, together with further genotyping of larger sample sets, reveals that all the Oirat-speaking populations studied here, including Kalmyks, share, as a dominant paternal lineage, Y-chromosomal haplogroup C3c1-M77, which is also present in several geographically distant native Siberian populations. We identify a subset of this clade, C3c1b-F6379, specifically enriched in Kalmyks as well as in Oirat-speaking clans in Inner Asia. This sub-clade coalesces at around 1500 years before present, before the Genghis Khan era, and significantly earlier than the split between Kalmyks and other Oirat speakers about 400 years ago. We also show that split between the dominant hg C variant among Buryats—C3-M407—and that of C3-F6379, took place in the Early Upper Palaeolithic, suggesting an extremely long duration for the dissipation of hg C3-M217 carriers across northern Eurasia, which cuts through today’s major linguistic phyla.

## Introduction

Kalmyks are the only Mongolic-speaking people living in Europe, residing in the easternmost part of the European Plain. According to historical evidence, the ancestors of Kalmyks were nomadic groups of Oirat-speaking people, who migrated from Western Mongolia to Eastern Europe about four centuries ago [1, 2]. Kalmyks settled down in the dry steppe area, west of the lower Volga River basin on the Northwest shore of the Caspian Sea.

**Fig. 1:**
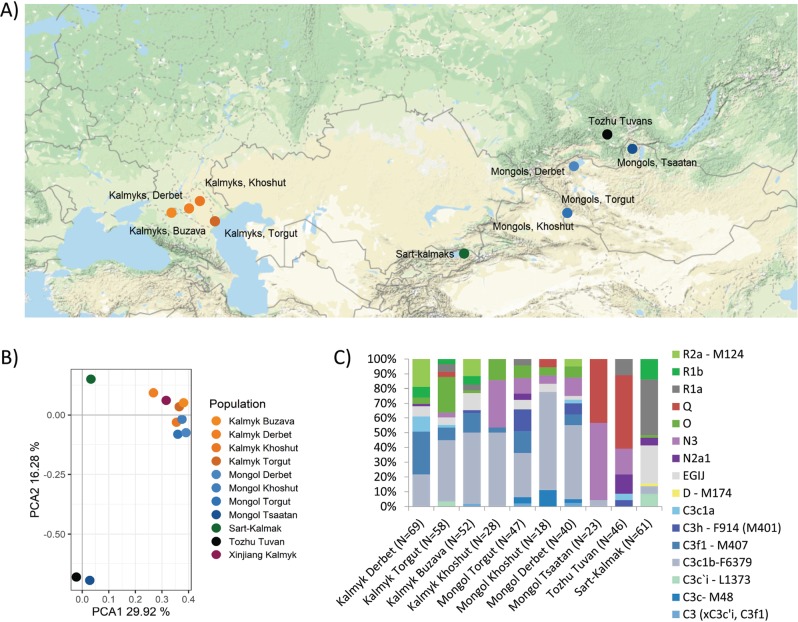
**a** Map with the sampling points of the studied populations. Kalmyk students from Xinjiang province in China were left out due to small sample size (<15). Mongol Torguts and Mongol Khoshuts live in the same location and are indicated with one dot. The map was created using ggmap package in software environment R [50]. Map data is from **c** 2018 Google Maps. **b** PC plot based on haplogroup frequencies in studied populations. **c** Bar chart of Y-chromosomal haplogroup distribution among the studied populations

Oirat dialects belong to the western branch of the Mongolian language family [3], whose speakers include numerous sub-ethnic groups (Derbet, Torgut, Khoshut, Olot, Dzungar (Zunghar), Bayad, Zakhchin, Khoton, Myangad, Buzava) across a wide geographical area of Uvs and Khovd provinces (aimags) of Western Mongolia (*N* = 209,412 [4]), and in Xinjiang Uygur Autonomous Region, China (*N* = 194,891 [5]). Ethnic groups of Oirat speakers in the Republic of Kalmykia, Russia (*N* = 162,740 (http://www.gks.ru/free_doc/new_site/perepis2010/croc/perepis_itogi1612.htm)) include Torguts, Derbets and Buzavas (Fig. 1a), together with a smaller group called Khoshuts, who live in just two villages of Kalmykia. Up until today the Kalmyks have retained their distinguished sub-ethnic groups, being quite separated from their geographical neighbours in Russia and northeast Caucasus.

Within the Torguts of Kalmykia the Tsaatan people form a sub-group, whereas in Mongolia Tsaatans are considered a distinct group on their own. Historically, Mongolian Tsaatans lived together with Tozhu Tuvans who speak a dialect of Tuvan, which is a Turkic language. They also shared a subsistence pattern, as both were reindeer herders. Researchers often erroneously consider the two Tsaatan groups together, relying on the similarity of ethnonyms and ethno-cultural characteristics [6]. The genetic relatedness of the two Tsaatan groups is therefore of great interest and has not been characterized thus far.

Kalmaks are another population of Oirat origin residing in small groups on the migration routes of Oirats through Altai and Central Asia. The largest group of Kalmaks is the Sart-Kalmak people living in Kyrgyzstan [7], who speak the Oirat dialect of the Mongolian language and profess adherence to the Islam faith. Relating their paternal genepool to that of the Kalmyk people could reveal the extent of potential common paternal genetic legacy.

In the Great Mongol Empire, the ruling dynasties of Oirats entered into matrimonial alliances with the dynasty of Genghis Khan [8]. Having a privileged position, they retained their tribal structure and were released from the need to pay tribute. The collapse of the Mongol Empire was followed by the formation of the Durben-Oirat alliance, which existed from the fourteenth to the eighteenth century [9]. The genealogical connection of Kalmyk Khoshut rulers to the younger brother of Ghenghis Khan (Habutu Hasar) [10] is mentioned in various documents (e.g., ‘*Tale of the Derben-Oirats*’ composed by Buddhist monk Gaban Sharab (1737)) and ideal for investigation using the Y chromosome.

Previous studies of the paternal genetic legacy of Genghis Khan and his male descendants [11–16] identified a C3*- Star Cluster [16]. However, a recent study by Wei et al. [17] suggests that the age of the most recent common ancestor (TMRCA) of the Star Cluster (proposed to be the Y-profile of Genghis Khan) and its sub-lineages, together with their expansion patterns, are more consistent as having resulted from the diffusion of Mongolic-speaking populations. Hence, there is a need for direct genotyping of additional, well-documented, male descendants from a wider geographic region, to investigate the paternal legacy of Genghis Khan and his descendants with greater precision.

Previous studies of the Y chromosome diversity among Kalmyks are limited by a lack of sub-ethnic differentiation among samples [18], or being limited to just one of the smallest sub-groups, the Khoshut [19], and the large sub-ethnic group of Buzava [20, 21] was not sampled in any of the genetic studies thus far. Here we study 454 Oirat-speaking individuals from Kalmykia (all 4 ethic groups), Mongolia (4 groups including Torguts and Tsaatans) and Kyrgystan (Sart Kalmaks), together with 28 Tozhu Tuvans from the Russian Federation. A combination of genotyping, microsatellite analysis and Y chromosome sequencing provides high-resolution comparative data between these geographically distant Oirat-speaking groups.

We have set the following objectives in our study: (a) to characterize the patrilineal genetic structure of the sub-ethnic groups of Kalmyks; (b) to determine the relation of Kalmyks to Oirat groups in Western Mongolia; (c) to clarify the controversial issues of ethnogenesis of the groups of Sart Kalmaks of Kyrgyzstan and the Tsaatans of Mongolia; (d) to show whether the members of the Khoshut dynasty in Kalmykia, whose genealogy traces back to Habutu Hasar (Genghis Khan’s younger brother) belong to the putative Genghis Khan Y-chromosomal lineage.

## Materials and methods

### Samples

We studied a total of 454 unrelated male individuals for Y-chromosome polymorphisms. Included samples were from four sub-populations of Kalmyks from Russian Federation: Torgut (58), Derbet (69), Buzava (52), Khoshut (28); 4 populations from Western Mongolia: Torgut (47), Derbet (40), Khoshut (18), Tsaatan (23); Sart Kalmaks from Kyrgyzstan (61); Kalmyk students from Xinjiang province in China (12); and Tozhu Tuvans from Russian Federation (46) (Fig. 1a). The study was approved by the Federal State Budgetary Institution Research Centre for Medical Genetics Moscow, Russia. All donors provided informed consent forms that were translated into the native languages of the donors. All experiments were performed in accordance with the relevant guidelines and regulations of the collaborating institutions. DNA from peripheral blood leukocytes was isolated using standard phenol chloroform method.

### Genotyping and data analyses

The following Y-chromosomal biallelic markers were genotyped: M9, M130, M217, M77, F6379, B469, B80, B90, L1373, M407, M217, M48, M401, М207, Page07, Z93, Z95, Z2125, M343, M269, M412, Z2105, М458, М558, M478, M124, M175, M122, M119, M268, M95, P203, P201, M7, M231, P43, TAT, M128, F2930, F4205, CTS6967, B478, B525, B187, M2118, YAP, M174, M35, M201, P15, M170, M423, 12f2, M410, Page08, M242, M25 and M346. Genotyping was performed using PCR and subsequent direct sequencing or restriction fragment length polymorphism analysis. For genotyping new markers (F6379, B80 and B469) discovered from the refined phylogenetic tree inside C3c1 sub-clade, we designed primers with Primer3 software [22, 23]. Primer specificity was first assessed with Primer-BLAST [24] and GenomeTester v.1.3 software [25], and verified by Sanger sequencing. The specifications for the new markers inside C3c1 sub-clade can be found in Supplementary Table S1.

We used R to perform the principal component analysis (PCA) [26] (Fig. 1b, Supplementary Figs. S1 and S2) and correspondence analysis (CA) [27] (Supplementary Fig. S3 and Supplementary Table S2). To obtain the pairwise Fst genetic distances between groups and to perform the analysis of molecular variation (AMOVA), we used Arlequin 3.5.1.2 [28] (Supplementary Tables S3 and S4).

### Microsatellites and phylogenetic network

The microsatellite analysis was performed for 78 samples using 23 Short Tandem Repeats (STRs) from PowerPlex Kit: DYS19, DYS385a, DYS385b, DYS389 I, DYS389 II, DYS390, DYS391, DYS392, DYS393, DYS437, DYS438, DYS439, DYS448, DYS456, DYS458, DYS481, DYS533, DYS549, DYS570, DYS576, DYS635, DYS643 and YGATAH4 (Supplementary Table S5).

We combined the data of 17 Y-STRs from 50 hg C3c1-M77 samples from this study (Supplementary Table S5) and hg C3c1-M77 samples published previously [19], to construct the phylogenetic network for hg C3c1 (Supplementary Fig. S4). We applied the median joining algorithm in the Network 4.6.1.1 software [29]. The expansion time calculations of the central node in the network were performed for 17 STRs using the rho-statistic and the genealogical mutation rate ~8.6 × 10^−5^ per locus per year for the Y-filer (discussed in ref. [30]).

### Whole Y chromosome sequencing

To reconstruct the phylogeny of haplogroup C3 we combined the sequences of 10 novel Y chromosomes with 28 published by us earlier [31] (Supplementary Table S6). The ten whole Y chromosome sequences reported in this study are deposited in the European Nucleotide Archive (http://www.ebi.ac.uk/ena) under the accession number PRJEB27561. Variants that did not have previously reported rs numbers were submitted to NCBI dbSNP database (https://www.ncbi.nlm.nih.gov/projects/SNP/) (dbsnp official release version 153).

All novel samples were sequenced using the Illumina HiSeq 2500 platform following Y chromosome capture with a proprietary capture protocol available at Gene by Gene (Family Tree DNA) using the commercially available “BigY” service (https://www.familytreedna.com/), a targeted enrichment design utilizing 67,000 capture probes for sequencing at least 10 Mbp at > 60 × coverage. The targeted regions lie within the non-recombining male-specific parts of the Y chromosome. Published genomes are generated with Complete Genomics (San Jose, California) technology at a mean coverage of 40×.

### Mapping and calling the Y chromosome variants

The fastq files of the newly sequenced genomes were mapped with BWA-MEM (v0.7.12) [32]. Read duplicates were removed using Picard (v2.0.1) (http://broadinstitute.github.io/picard/). GATK (v3.5) [33] was used to perform local realignment around known indels and for base quality score recalibration. Variant calling was performed with GATK tools HaplotypeCaller and GenotypeGVCF, which were asked to report variant and non-variant calls over the whole Y chromosome.

### Filtering

Individual genotypes were filtered with bcftools (v1.4) [34] in the raw VCF files that included all Y-chromosomal sites. The filtered Illumina and Complete Genomics data sets were merged using CombineVariants from GATK (v3.8) [33]. All the positions with >5% of missing genotypes in the combined dataset were masked out resulting in the final effective overlap between the sets. In addition, regions with poor mapability as described in ref. [31] were also masked out. This resulted in a final total of 9.2 Mb of usable sequence.

### Phylogeny reconstruction

**Fig. 2:**
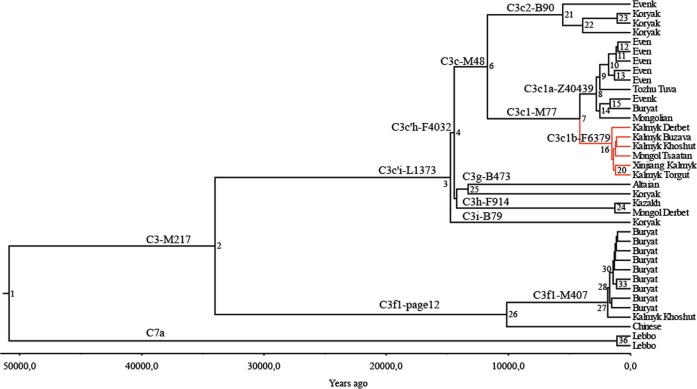
Detailed phylogenetic tree of hg C3. A phylogenetic tree of hg C3 based on 37 high-coverage Y chr sequences. As an outgroup, two hg C7a sequences were used. The calibrated tree was constructed using BEAST v.1.7.5 software package. Internal nodes, sub-clade names and population names on the tips are indicated. Internal nodes with posterior probabilities <0.73 are not shown. Newly characterized C3c1b-F6379 branch is indicated in red. Age estimates can be found in Supplementary Table S7. All the sub-clade (node) defining variants and marker names are reported in Supplementary Table S8

We reconstructed the phylogeny for Y chromosome haplogroup C3 with hg C7a as an outgroup and estimated coalescent times using the software package BEAST v.1.7.5 [35] (Fig. 2). We chose Bayesian skyline coalescent model as the tree model [36] the general time reversible substitution model [37] with γ-distributed rates [38] and a relaxed lognormal clock model [39]. We used previously published [31] age of hg C (50,865 years, 95% confidence interval = 49,191–52,699) as the calibration point to get coalescent times for the inner structure. The run was performed with a piecewise-constant coalescent model with seven groups. The number of groups was obtained by dividing the sample size by 5. Marcov Chain Monte Carlo method had 30 million iterations with a sampling made every 2000 steps. We visualized the BEAST run in Tracer v1.5 (http://beast.bio.ed.ac.uk) and confirmed that effective sample size was above 200. The tree was visualized using FigTree 1.4.2. (http://tree.bio.ed.ac.uk/software/figtree/). One newly sequenced sample was left out of BEAST analysis due to quality issues but can be seen on annotated tree indicated with dashed line (GRC171156839) (Supplementary Fig. S5).

Throughout the study, nomenclature of Karmin et al. [31] and its updates were followed.

## Results and discussion

When considering all the studied populations together, >50% of the samples belong to different branches of haplogroup С3. If patrilineally dissimilar groups of Turkic-speaking Tsaatans and Tozhu Tuvans, and Oirat-speaking Sart Kalmaks are excluded, then the share of C3 becomes 62.5% among the sub-populations of Kalmyk and Oirats from Mongolia.

**Fig. 3:**
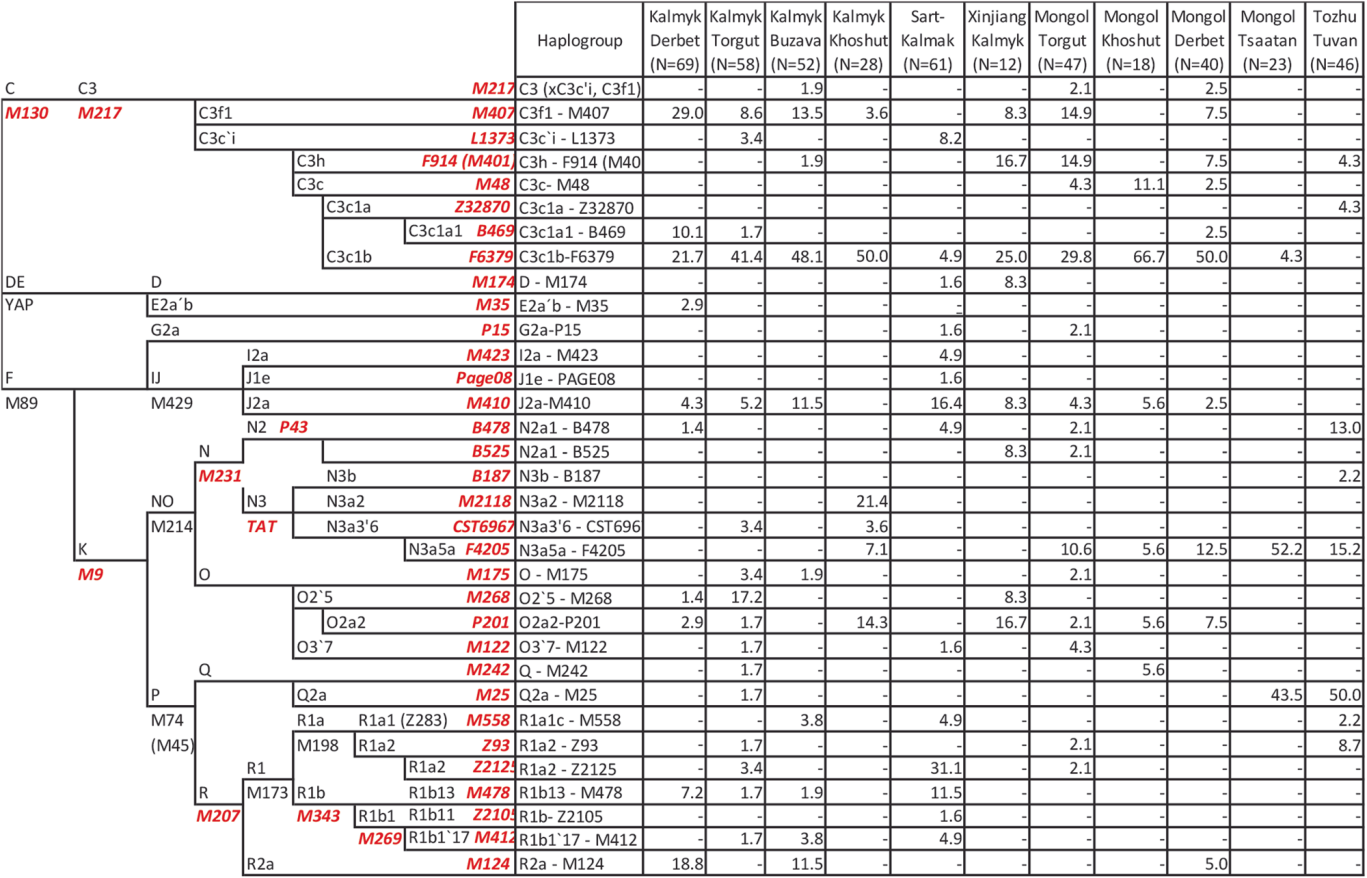
Schematic phylogenetic tree of detected Y SNPs and distribution of Y chromosome haplogroup frequencies (%) in the studied populations. Markers typed are indicated in red

The new phylogeny for haplogroup C3c1-M77 based on Y-chromosome whole sequences (Fig. 2) reveals two sub-branches: (i) previously characterized C3c1a-Z40439, which is common among Tungusic-speaking Evens and Evenks, Mongolic-speaking Buryats and Mongols [31], and (ii) a novel sub-branch that we name C3c1b-F6379, after 1 of 15 shared variants. The Bayesian estimate of the time to TMRCA of C3c1b is ~1.5 thousand years ago (KYA) with the 95% highest posterior density limit interval of 1.1–2.1 KYA (Table S7). The genotyping of the F6379 marker demonstrates that C3c1b is the most common paternal sub-clade among Kalmyks and Oirats from Mongolia, comprising 40.3% of male lineages (Fig. 1c and Fig. 3). It occurs at minor frequencies among Mongol Tsaatans and Sart Kalmaks.

The genotyped samples from this study belong to 33 Y-chromosomal haplogroups. The frequency distribution among the studied populations is shown in Fig. 1c and Fig. 3. Genotyping results indicate that Kalmyk sub-groups are quite diverse. This could be because different Kalmyk sub-groups experienced varying amounts of admixture after their migration from Mongolia. However, the evidence from paternal lineages suggests that Kalmyks have existed in relative isolation since arriving in Russia and maintained their common genetic heritage due to religious and cultural differences with their geographic neighbours in Eastern Europe. This scenario is also consistent with ethnographic evidence indicating that their current sub-ethnic groups were formed through the merging of different smaller tribal groups in the past [9, 40]. In contrast, Tozhu Tuvans and Mongol Tsaatans have only a limited number of haplogroups, typical of a founder event followed by isolation and small census size (Fig. 1c).

Although sub-lineages of hg C3 form the major shared component among the various sub-populations of Kalmyks and Mongolian Oirat sub-groups, the frequency and composition of other haplogroups differ (Fig. 3 and Fig. 1b). In addition to C3, the other Y chromosome haplogroups present in Kalmyks are primarily of Siberian or Eastern Asian origin (hg N, O, Q, R1a2-Z93 derivates, R2). For example, haplogroup N occurs in Kalmyk Khoshuts more frequently (32.1%) than in the other studied populations. N3a2-M2118 (Fig. 3), which is common in Yakutia (Central Siberia) and less frequent in Khanty and Mansi [41], is carried by 21.4% of Kalmyk Khoshuts (Fig. 3), who also have 7.1% of N3a5a-F4205, usually found in Buryats and Mongols [41], and 14% of O2a2-P201, which occurs at a high frequency in China and Southeast Asia [42].

Sart Kalmaks, Tsaatans of Mongolia and Tozhu Tuvans diverge from the general haplogroup distribution pattern (Fig. 1b, Fig. 1c and Fig. 3). Sart Kalmaks deviate due to >50% frequency of hg R. The most frequent (>30%) sub-group of hg R among Sart Kalmaks is R1a2-Z2125, which is at high frequencies in Kyrgyzstan and is also present in numbers among the Afghan Pashtuns [43]. This strongly suggests male-mediated gene flow between Kyrgyz and Sart Kalmaks reflected in small cultural and phenotypic differences between these neighbouring populations. The Tsaatans of Mongolia, with two major Y-chromosome lineages (N3a5a-F4205 and Q1a1b-M25) constituting ~96% of the paternal genepool, are another group with evidence for a founder effect followed by genetic isolation, typical of a small population of taiga reindeer herders. The same two lineages also dominate in the Tozhu Tuvans (N3a5a-F4205 (15.2%) and Q1a1b-M25 (50.0%)), providing genetic confirmation of inter-marriage between the two groups before the establishment of a political border between the Tuva Republic and the Russian Federation in 1944) [44]. According to the distribution of previously published Y chromosome haplogroup frequencies, Mongolian Tsaatans and Tozhu Tuvans are also similar to Tuvans [45]; hence, all three likely share a degree of common paternal origin.

Despite isolation by distance for four centuries, the distribution of Y-chromosomal haplogroups among Kalmyk sub-groups and Oirats of Western Mongolia is quite similar (Fig. 1c and Fig. 3). This similarity in paternal genepool composition between Kalmyks and Oirats from Mongolia is clearly visible in the PCA plot (Fig. 1b) where they cluster tightly together, whereas Kalmyks and their near geographical neighbours (NE Caucasus, Central and Southern Russians, Tatars and Kazakhs) [46–48] are far apart (Supplementary Fig. S1). According to pairwise genetic distances (Fst), Mongol Tsaatan, Tozhu Tuvan and Sart Kalmak exhibit statistically significant divergence from the remaining studied populations (Supplementary Table S3). Repeating the PCA without these three outliers highlights the displacement of Kalmyk Derbet (Supplementary Fig. S2), which is driven by the significant level of haplogroup R2a (CA Dim1 = 21.67) (Fig. 3, Supplementary Fig. S3 and Supplementary Table S2). According to CA, other strong drivers of differentiation are N3a2-M2118 (Dim2 = 19.22) (Supplementary Fig. S3 and Supplementary Table S2), only present in Kalmyk Khoshuts, and O2′5-M268 (Dim2 = 33.9) (Supplementary Figure S3 and Supplementary Table S2) with the highest frequency among Kalmyk Torguts. In addition to Kalmyk Derbet, the genetic distances separating Kalmyk Buzava from rest of the groups are also statistically significant (Table S3). The AMOVA results confirm the pattern seen on the PC plot (F_CT_ = 0.15670, *P* = 0.00293) (Supplementary Table S4). The AMOVA results also show a low (F_CT_ = 0.01771) and insignificant (*P* = 0.13196) level of differentiation among the geographically distant groups (Supplementary Table S4). The parsimonious explanation for this concordance is that Y-chromosome genetic structure of sub-groups of Oirat-speaking Mongols developed in the territory of Western Mongolia and did not undergo significant changes after the split, neither among Kalmyk people nor in Oirat branches in Inner Asia.

Returning to the C31b lineage, the STR network (Supplementary Fig. S4) shows a supercluster with a strongly centred star-like pattern and men from different sub-populations of Kalmyks and Oirats from Mongolia share the central modal haplotype, suggesting a recent shared ancestry and strong founder effect. The STR haplotype of putative descendant of Genghis Khan’s male lineage is two mutational steps away from the central node. The expansion time for the central node of the network, calculated with the rho-statistic and the mutation rate for STRs (discussed in ref. [30]), is 667 (±155) years, arguably giving support to the idea of this haplotype expanding with the Mongol conquests. This time estimate, however, is significantly younger than the estimation based on sequence data at 1076–2011 years (Supplementary Table S7 and Fig. 2, node 16).

The differences in TMRCA estimates based on network analysis of Y-STRS vs. BEAST analysis of sequences may be caused by using different methods and different datasets. Non-random selection of samples for sequences to cover as much variation as possible provides the maximum TMRCA estimate for the clade and rho-statistic calculations based on STR network give us the time estimate for the pronounced founder event in the populations. It should be noted that the mutation process of microsatellites is less well understood and repeat length changes can occur in both directions, leading to underestimation of times.

## Conclusions

Although it has been previously shown by Nasidze et al. [31] that both Y chromosome and mtDNA hgs display a close contact between Kalmyks and Mongolians, our higher resolution analyses of patrilineal population structure of Mongolian Oirats and Kalmyks in the Russian Federation, to our knowledge, show for the first time the unity and integrity of the paternal gene pools of Kalmyk and Mongolian Oirat sub-groups. In particular, the Derbets and Torgut ethnic groups have similar genetic profiles, despite being separated by thousands of kilometres for around 400 years, whereas there exists a clear genetic and cultural continuity between the various Kalmyk groups in the Russian Federation.

However, the ethnogenesis of the Sart Kalmaks of Kyrgyzstan and the Tsaatans of Mongolia, appears to have a somewhat different history. The Sart Kalmaks show a more limited relatedness to the Oirat-speaking groups (having only ~13% of hg C3c) together with evidence of significant paternal gene flow, likely from neighouring Kyrgyzs, whereas the Tsaatans of Mongolia demonstrate closer genetic affinities with Tuvinians and Tozhu Tuvans.

The evidence presented here shows that the lineage of the putative Koshut rulers with possible genealogical links to the brother of Genghis belongs to haplogroup C3-M77. Based on the aDNA findings of the putative members of Genghis Khan dynasty, Zhang et al. [49] speculate that their paternal lineage may be within C3-L1373 lineage. However, it is widespread all over northern Siberia, from Beringian Koryaks to southern Siberian Altaians and Kazakhs (Fig. 2). Sub-clades, such as M77, seem to be present more typically among Mongolic- and Tungusic-speaking populations. The present study highlights a much more recent derivative of it, C3-F6379 that is typical for Kalmyks and Oirats from Mongolia. However, it is also clear that the patrilineal identity of Genghis Khan clan can be reliably identified by ancient DNA study of the archaeologically authentic remains belonging to this imperial family that so far have not been found.

## Supplementary information

Figure S1

Figure S2

Figure S3

Figure S4

Figure S5

Table S1

Table S2

Table S3

Table S4

Table S5

Table S6

Table S7

Table S8

Supplementary legends
