# Supplementary figures and images for: Y-chromosomal analysis of clan structure of Kalmyks, the only European Mongol people, and their relationship to Oirat-Mongols of Inner Asia
Source: Eur J Hum Genet. 2019 Apr 11;27(9):1466–74. doi: 10.1038/s41431-019-0399-0 (PMC6777519; doi:10.1038/s41431-019-0399-0)

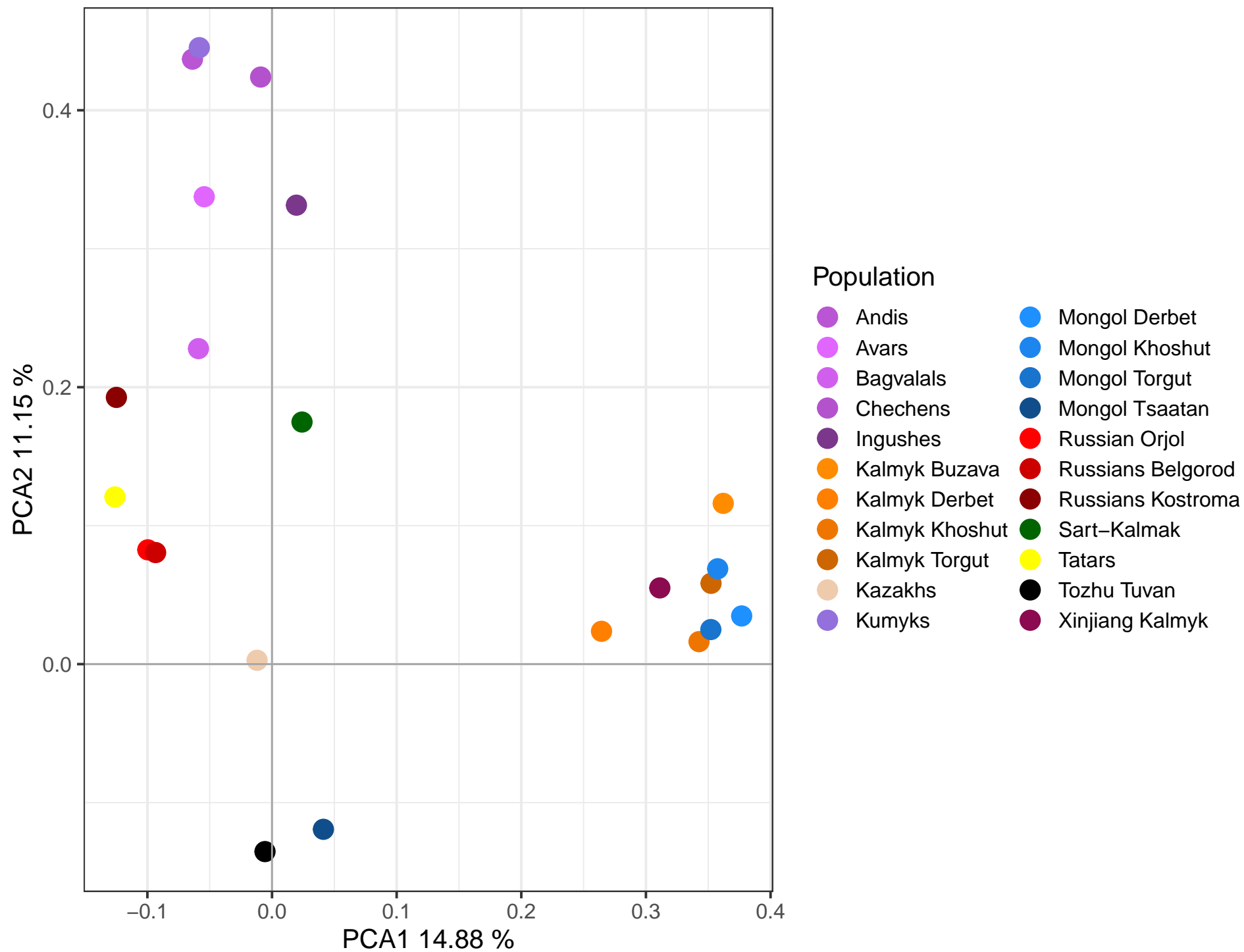

Supplement: Supplementary file 1 — Figure S1 [file 41431_2019_399_MOESM1_ESM.pdf]

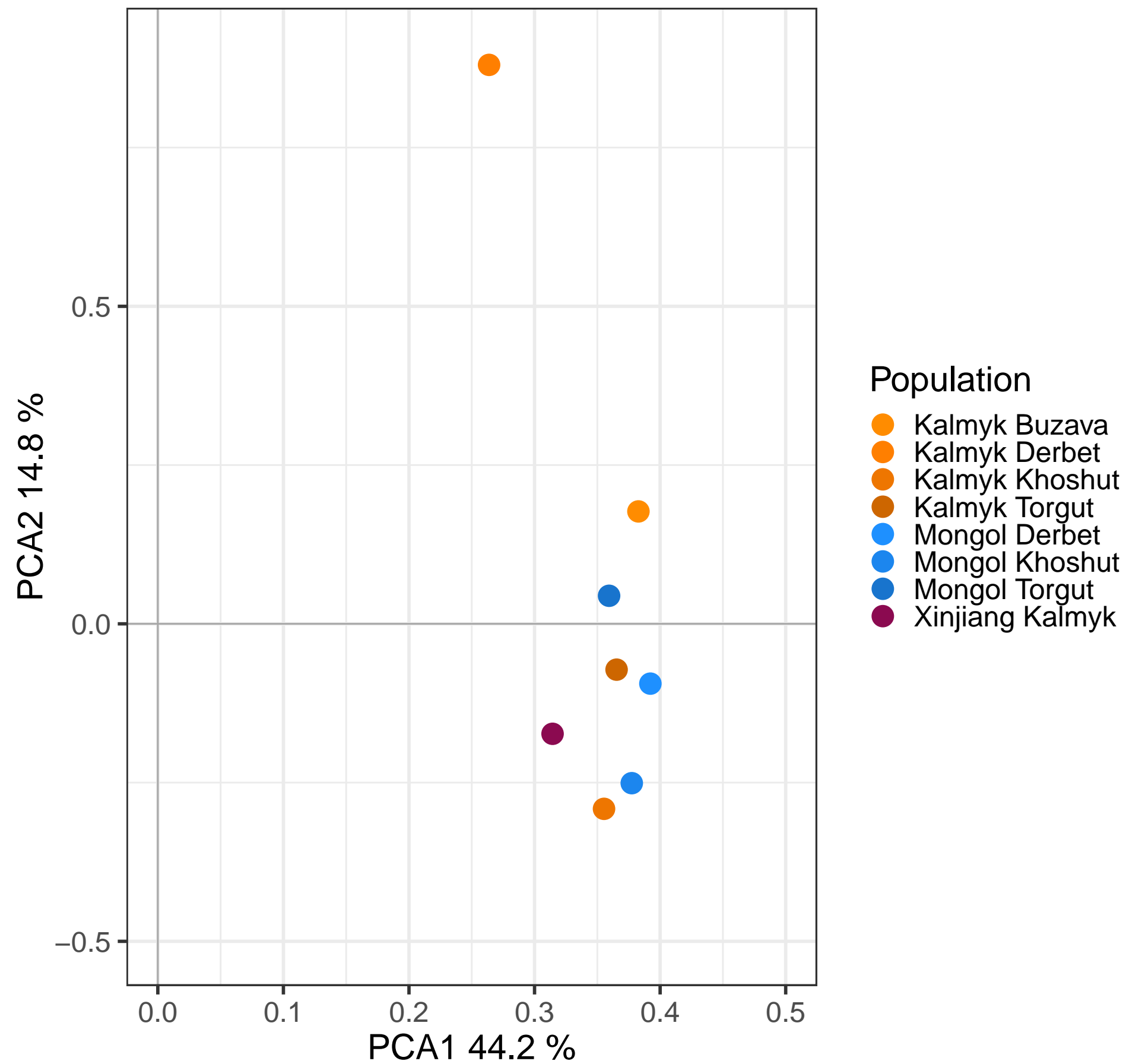

Supplement: Supplementary file 2 — Figure S2 [file 41431_2019_399_MOESM2_ESM.pdf]

CA – Biplot

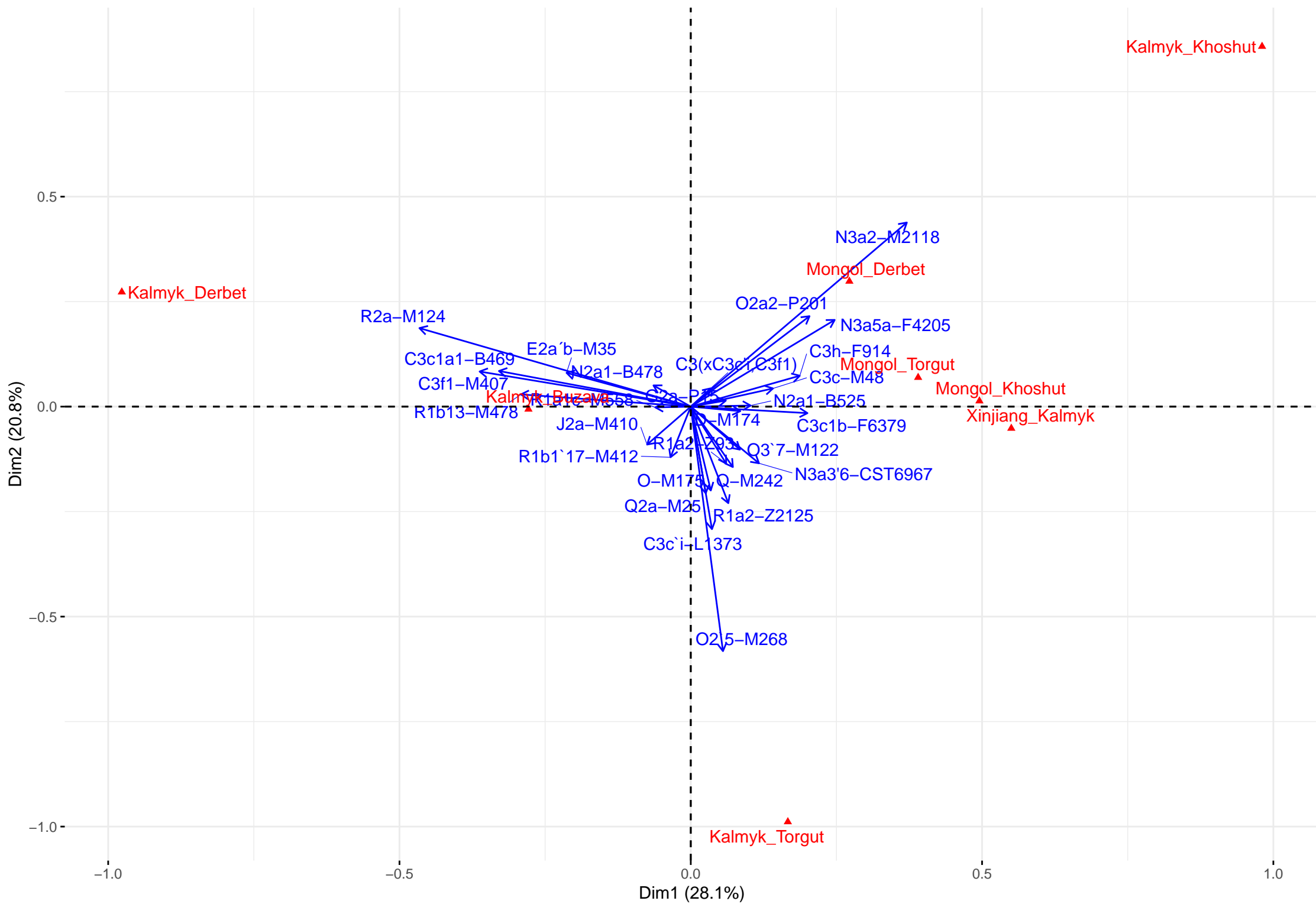

Supplement: Supplementary file 3 — Figure S3 [file 41431_2019_399_MOESM3_ESM.pdf]

# Geography

Mongols

Kalmyks

Sart-Kalmaks

Tuva Todga

Putative Genghis Khan  
descendant

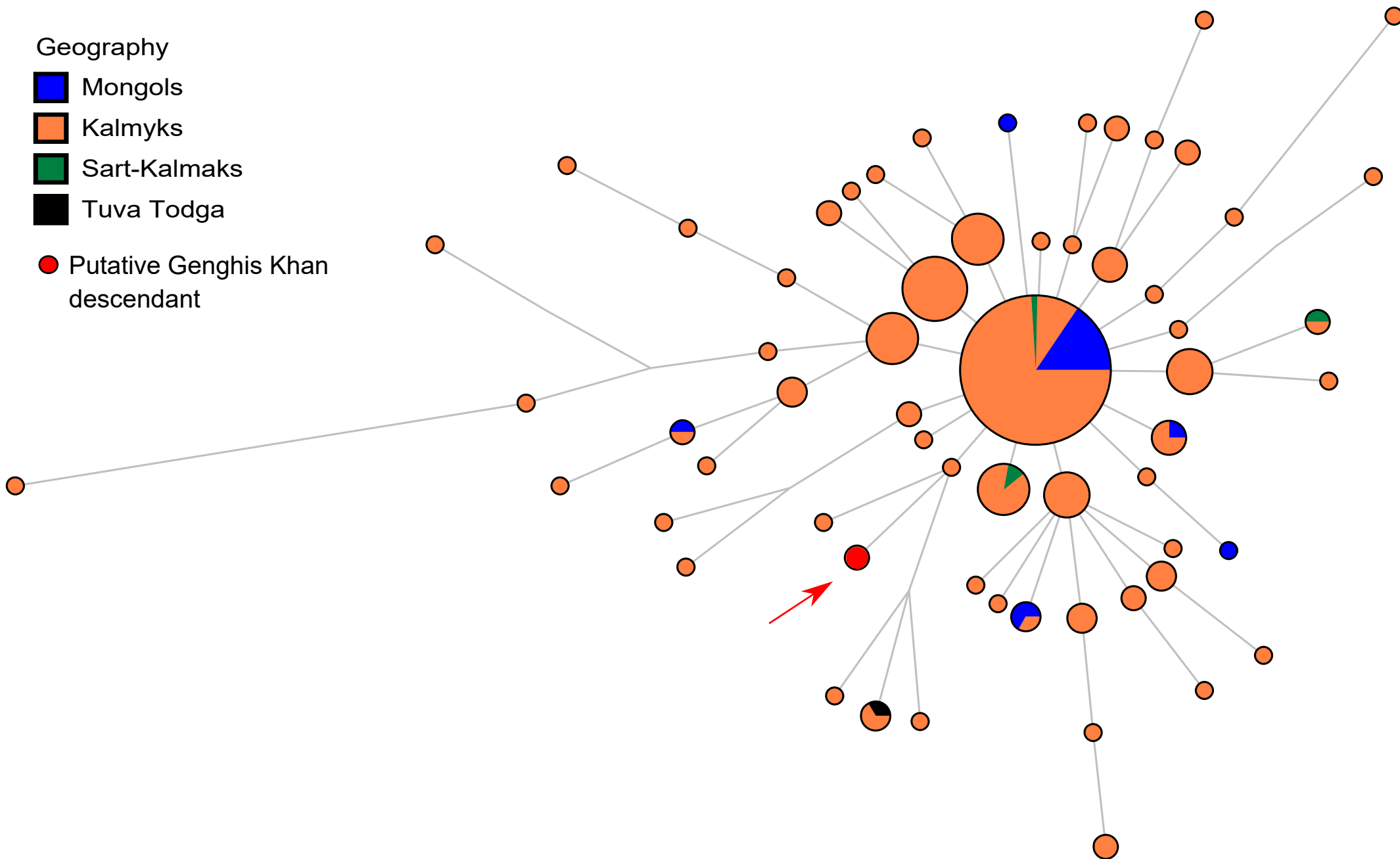

Supplement: Supplementary file 4 — Figure S4 [file 41431_2019_399_MOESM4_ESM.pdf]

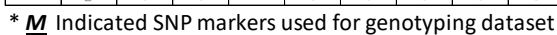

Supplement: Supplementary file 5 — Figure S5 [file 41431_2019_399_MOESM5_ESM.pdf]
