# Supplementary material for: Y-chromosomal analysis of clan structure of Kalmyks, the only European Mongol people, and their relationship to Oirat-Mongols of Inner Asia
Source: Eur J Hum Genet. 2019 Apr 11;27(9):1466–74. doi: 10.1038/s41431-019-0399-0 (PMC6777519; doi:10.1038/s41431-019-0399-0)
Supplement: Supplementary file 14 — Supplementary legends [file 41431_2019_399_MOESM14_ESM.docx]

**Supplementary**

Figures include two PC plots of haplogroup frequencies, Y chromosome haplogroup contributions based on the CA, the median network of C3c1-M77, and the annotated tree of C3. Tables are Excel files including specifications of new markers, Y chr hg contributions based on CA, Fst distances, AMOVA results, Y-STR’s, list of high coverage samples, age estimates of C3 clades, and branch-defining variants.

**Figures:**

**Figure S1.** PC plot based on haplogroup frequencies in studied populations and their geographic neighbours.

**Figure S2.** PC plot based on haplogroup frequencies in studied populations without the three outliers (Sart-Kalmak, Tozhu Tuvan and Mongol Tsaatan).

**Figure S3.** Contribution biplot based on haplogroup frequencies without three PCA outliers (Sart-Kalmak, Tozhu Tuvan and Mongol Tsaatan).

**Figure S4.** The median network of C3c1-M77. Node size is concordant with the frequencies of haplotypes and colour code is indicated on the figure. Samples used are from this study and literature [19].

**Figure S5.** Manually annotated tree of hg C3.

**Tables:**

**Table S1.** Specifications for new markers inside C3c1 used to genotype population samples

**Table S2.** Y chromosome haplogroup contributions to 2 dimensions based on the correspondence analysis.

**Table S3.** Fst distances based on haplogroup frequencies

**Table S4.** AMOVA results for studied populations based on Y-chromosome haplogroup frequencies

**Table S5.** Y-STR haplotypes of C3 individuals included in networks

**Table S6.** List of high coverage samples and their geographic affiliations

**Table S7.** Age estimates of hg C3 clades

**Table S8**. Branch-defining variants and their correspondence to the nodes
